# Comparative analysis of success rates and complications between lumbar microdiscectomy and open discectomy for lumbar disc herniation

**DOI:** 10.12669/pjms.41.4.10620

**Published:** 2025-04

**Authors:** Zahid Khan, Seema Sharafat, Haider Ali

**Affiliations:** 1 Zahid Khan, FCPS, MTI, Lady Reading Hospital, Peshawar Pakistan; 2 Seema Sharafat, FCPS, MTI, Lady Reading Hospital, Peshawar Pakistan; 3 Haider Ali, FCPS, MTI, Lady Reading Hospital, Peshawar Pakistan

**Keywords:** Complications, Lumbar microdiscectomy, Lumbar disc herniation, Open discectomy, Success rates

## Abstract

**Objective:**

This study aimed to evaluate the success rates and complications associated with lumbar microdiscectomy compared to open discectomy from a tertiary care hospital perspective.

**Methods:**

A retrospective analysis was conducted at the Neurosurgery Department of Lady Reading Hospital over four years (January 2020 to December 2023). Inclusion criteria comprised patients of all genders and ages who underwent single-level lumbar microdiscectomy or open discectomy. Exclusion criteria encompassed patients who underwent redo surgeries, or had incomplete data.

**Results:**

In the study, 396 patients were enrolled, with 153 undergoing open discectomy and 243 undergoing microdiscectomy. The mean age of the participants was 40.42±11.49 years. Noteworthy findings from the analysis include that the success rates were 77.8% for open discectomy and 86.8% for microdiscectomy (p=0.050), while complications, specifically discitis, occurred in 2.6% of open discectomy patients and 0.4% of microdiscectomy patients (p=0.025).

**Conclusion:**

Microdiscectomy exhibited higher success rates and lower complication rates than open discectomy for single-level lumbar disc herniation, underscoring its potential as a preferred surgical intervention.

## INTRODUCTION

Lumbar disc herniation occurs when the nucleus pulposus of the intervertebral disc protrudes through the fibrous annulus, often in the posterolateral area.^1^ This displacement can compress and irritate the lumbar nerve roots and dural sac, resulting in sciatica. While sciatica has been known since ancient times, its link to disc herniation was first identified by Mixter and Barr in the early 20th century.^2^ Presently, lumbar disc herniation is the most prevalent diagnosis among degenerative lumbar spine disorders and a leading cause of spinal surgery.^3^ Increased access to medical care, the routine use of imaging examinations, and the safety of surgical interventions have contributed to the high incidence of surgical treatment despite the condition often being self-limiting.

The treatment modalities for lumbar disc herniation have progressed from transdural resection to conventional open approaches,^4^,^5^ followed by the introduction of microsurgery, endoscopic, and percutaneous techniques. Open lumbar discectomy has long been considered the gold standard for surgical treatment.^6^ Although conservative therapy alleviates symptoms in most cases, 10-20% of patients experience persistent pain that necessitates surgical intervention.^7^,^8^ Open lumbar discectomy, despite potential complications such as scar tissue formation, facet joint damage, and postoperative lumbar instability, remains widely practiced due to its favorable clinical outcomes.^9^,^10^

The pioneering work of Mixter and Barr paved the way for Microdiscectomy operations,^11^ which emerged in the 1970s with the advent of surgical microscopy. Microdiscectomy has since become the standard treatment for lumbar disc herniation, offering advantages such as reduced tissue damage compared to conventional open discectomy, resulting in less epidural fibrosis, postoperative pain, and segmental instability.^12^,^13^ Recent advancements have led to the development of microendoscopic discectomy, a minimally invasive variation of microdiscectomy. While microsurgery and sequestrectomy may demonstrate early-stage advantages in selected cases, long-term outcomes often reveal no significant differences.^14^

The utilization of microscopy enhances the visual field, illumination, and differentiation of neural tissues and disc material. Despite advancements in endoscopic and microscopic techniques, Open discectomy remains valued for its extensive accumulated experience, absence of the need for specialized equipment, and shorter learning and operation times.^13^ The purpose of this study was to compare the success rates and complications of lumbar microdiscectomy versus open discectomy for treating lumbar disc herniation.

## METHODS

This retrospective cohort study was designed to compare the success rates and complications associated with lumbar microdiscectomy and open discectomy. The study was conducted at the Neurosurgery Department of Lady Reading Hospital, a tertiary care center, over four years, from January 2020 to December 2023.

### Ethical consideration:

Ethical Approval was obtained from the institutional review board (IRB) [Reference#; 216/LRH/MTI, Dated July 3, 2024]. Informed consent was waived due to the retrospective nature of the study, as it involved the analysis of pre-existing medical records.

### Inclusion Criteria:

- Patients of any gender and age who underwent single-level lumbar microdiscectomy or open discectomy.

### Exclusion Criteria:

- Patients who underwent revision (redo) surgery, as prior interventions could influence success rates and complication rates.
- Patients with incomplete medical records, as missing data may compromise the validity of the analysis.

### Variables:

The independent variable was the type of surgical procedure, i.e., microdiscectomy and open discectomy. The dependent variables included success rates and complications associated with these procedures.

### Data sources/Measurement:

Data were extracted from patient medical records over the specified period. Collected data included demographic information, clinical characteristics, surgical outcomes, and postoperative complications.To minimize bias, the study included consecutive patients who met the inclusion criteria during the specified timeframe. Selection bias was reduced by using explicit inclusion and exclusion criteria. Data abstraction was performed uniformly to maintain consistency.The study included 396 patients; 153 underwent open discectomy, and 243 underwent microdiscectomy.

### Statistical analysis:

The data was analyzed using SPSS version 25.0. Descriptive statistics were used to summarize the continuous and categorical variables as mean, standard deviations, frequency and percentages. Chi-square tests and Independent Sample T-tests were used to compare variables between groups, where a p-value < 0.05 was considered statistically significant.

## RESULTS

A comparison of demographic and clinical characteristics between patients undergoing open discectomy and microdiscectomy for lumbar disc herniation is presented in Table-I. Age distribution showed no significant difference between the two groups, with mean ages of 40.78 ± 11.02 years for open discectomy and 40.19 ± 11.80 years for microdiscectomy. However, the duration of operation differed significantly, with open discectomy patients having a longer duration (43.58 mins) compared to microdiscectomy patients (38.65 minutes), with a p-value of <0.01, indicating statistical significance. Additionally, no significant differences were observed in the distribution of symptoms such as leg warmness, leg weakness, or cauda equina syndrome. Regarding the affected side, there was a slight predominance of left-sided involvement in the microdiscectomy group compared to the open discectomy group. However, this difference was not statistically significant (p = 0.155). Analysis of the spinal levels involved revealed no significant differences between the two groups across various levels. Table-II overviews the success rates and complications observed in patients who underwent open discectomy or microdiscectomy for lumbar disc herniation. Regarding success rates, most patients experienced successful outcomes, with 83.3% in the open discectomy group and 86.8% in the microdiscectomy group.

**Table-I:** Comparison of demographic and clinical characteristics between open discectomy and microdiscectomy patients.

Variables		Total (N=396)	Procedure		P-value
			Open Discectomy (N=153)	Microdiscectomy (N=243)	
Age; years		40.42±11.49	40.78±11.02	40.19±11.80	0.621
Duration of operation; min		40.56±11.81	43.58±11.44	38.65±11.67	<0.01*
Gender	Male	241(60.9%)	95(62.1%)	146(60.1%)	0.690
	Female	155(39.1%)	58(37.9%)	97(39.9%)	
Symptom	LBP + Leg pain	351(88.6%)	132(86.3%)	219(90.1%)	0.453
	Leg Warmness	4(1.0%)	2(1.3%)	2(0.8%)	
	Leg Weakness	35(8.8%)	15(9.8%)	20(8.2%)	
	Cauda Equina Syndrome	6(1.5%)	4(2.6%)	2(0.8%)	
Pain Side	Left	240(60.6%)	86(56.2%)	154(63.4%)	0.155
	Right	156(39.4%)	67(43.8%)	89(36.6%)	
Spinal Levels	L1-2	4(1.0%)	1(0.7%)	3(1.2%)	0.649
	L2-3	11(2.8%)	2(1.3%)	9(3.7%)	
	L3-4	59(14.9%)	23(15.0%)	36(14.8%)	
	L4-5	176(44.4%)	71(46.4%)	105(43.2%)	
	L5-S1	146(36.9%)	56(36.6%)	90(37.0%)	

* p<0.05 is considered statistically significant.

**Table-II:** Success & Complications Between Open Discectomy and Microdiscectomy Patients.

		Total (N=396)	Procedure		P-value
			Open Discectomy (N=153)	Microdiscectomy (N=243)
Success	Deteriorated	14(3.5%)	8(5.2%)	6(2.5%)	0.050*
	Yes	330(83.3%)	119(77.8%)	211(86.8%)	
	No	23(5.8%)	14(9.2%)	9(3.7%)	
	Partial	29(7.3%)	12(7.8%)	17(7.0%)	
Complications	Discitis	5(1.3%)	4(2.6%)	1(0.4%)	0.025*
	Dural Tear	9(2.3%)	6(3.9%)	3(1.2%)	
	Hematoma	5(1.3%)	3(2.0%)	2(0.8%)	
	Infection	9(2.3%)	5(3.3%)	4(1.6%)	
	Neurological Complications	7(1.8%)	3(2.0%)	4(1.6%)	
	None	356(89.9%)	129(84.3%)	227(93.4%)	
	Spondylolisthesis	2(0.5%)	2(1.3%)	-	
	Wrong Level	3(0.8%)	1(0.7%)	2(0.8%)	

* p<0.05 is considered statistically significant.

However, a slightly higher proportion of patients in the microdiscectomy group experienced deterioration (5.2%) compared to the open discectomy group (3.5%) (p=0.050). Complications were relatively rare, with discitis being the most common, observed in 1.3% of open discectomy patients and 0.4% of microdiscectomy patients, with a significant difference noted (p=0.025). Dural tears, hematoma, infection, and neurology issues were also observed, albeit at low frequencies. The majority of patients did not experience any complications, with 89.9% in the total sample, 84.3% in the open discectomy group, and 93.4% in the microdiscectomy group. Spondylolisthesis was observed in 0.5% of the total sample, while wrong-level surgery occurred in 0.8%.

## DISCUSSION

In our study, the preoperative evaluations across both groups were consistent, revealing no disparities in baseline demographic and clinical characteristics except for the duration of the operation. Notably, the most commonly affected spinal segments were L4–5 and L5–S1 in both groups, aligning with findings from previous studies.^14^-^16^ A significant finding was the variation in operation duration between an open discectomy and a Microdiscectomy. On average, an open discectomy took 43.58 minutes, while a Microdiscectomy required 38.65 minutes. This is contrary to outcomes observed by Schmid et al., where the duration of an open discectomy was shorter, and hospital stays were significantly reduced with microdiscectomy.^14^

Regarding success rates, the majority of patients experienced positive outcomes, with 83.3% in the open discectomy group and 86.8% in the microdiscectomy group. Complications, though rare, included discitis, observed in 1.3% of open discectomy patients and 0.4% of microdiscectomy patients, with a significant difference noted (p=0.025). Most patients did not experience complications, with rates at 89.9% in the total sample, 84.3% in the open discectomy group, and 93.4% in the micro discectomy group. Comparisons with historical studies, particularly Caspar’s observation from 1977 emphasized the advantages of microdiscectomy, including reduced tissue damage, blood loss, bed rest, quicker mobilization, and return to work.^12^

Phan et al. reviewed extensive studies comparing full endoscopic discectomy, microdiscectomy, and open discectomy.^17^ The clinical outcomes indicated that full endoscopic discectomy and microdiscectomy had similar results to open discectomy, with no significant differences in complication rates. However, micro endoscopic discectomy demonstrated benefits such as less trauma to paravertebral tissue, reduced blood loss, shorter bed rest duration, and decreased persistent back pain.

In our study, the incidence of preoperative dural rupture was 3.9% for open discectomy and 1.2% for microdiscectomy, contrasting with literature values (open discectomy, 1.8%-2.7% and microdiscectomy, 3%-5.7%).^18^-^20^ This discrepancy suggests improvements in surgical techniques, particularly in microsurgical approaches. Wound infection rates were also noted, with open discectomy at 3.3% and microdiscectomy at 1.6%, aligning with findings from Schmid et al.^14^

Overall, this study offers important insights into the comparative effectiveness and safety of lumbar microdiscectomy versus open discectomy for treating lumbar disc herniation. Although the baseline characteristics of patients in both groups were similar, significant differences were found in operation duration and complication rates. Microdiscectomy had shorter operation times and lower complication rates, such as discitis, suggesting its potential advantages over open discectomy. These findings add to the growing evidence supporting microsurgical techniques for managing lumbar disc herniation. Future research about lumbar disc herniation management should explore long-term functional results, cost-effectiveness and satisfaction levels of patients to advance surgical decision-making.

### Limitations:

Despite the strengths of this study, several limitations must be acknowledged. First, its retrospective design may have introduced inherent biases and limitations in data collection. Second, the study was conducted at a single tertiary care center, which may limit the generalizability of the findings to other settings or populations. Additionally, the relatively short follow-up period might not have captured long-term outcomes and complications associated with the surgical interventions. Finally, the study did not evaluate patient-reported outcomes or quality of life measures, which are crucial for assessing the overall success of surgical interventions.

## CONCLUSION

Our findings indicate that microdiscectomy provides better outcomes in terms of operation duration and complication rates than open discectomy for lumbar disc herniation treatment. These results add to the ongoing discussion about the best surgical approaches for this common spinal condition. Further prospective studies with larger sample sizes and more extended follow-up periods are needed to confirm these findings and guide clinical practice.

### Authors’ Contribution:

**ZK:** Conception and design, acquisition of data, analysis and interpretation of data.

**SS:** Literature search, Drafting the article or revising it critically

HA: Literature search, Critical analysis,

All authors have approved the final version and are accountable for the integrity of the study.
